# Supplementary material for: Urban form and COVID-19 cases and deaths in Greater London: An urban morphometric approach
Source: Environ Plan B Urban Anal City Sci. 2022 Oct 14;50(5):1228–43. doi: 10.1177/23998083221133397 (PMC9574546; doi:10.1177/23998083221133397)
Supplement: Supplemental Material - Urban form and COVID-19 cases and deaths in Greater London: An urban morphometric approach [file sj-pdf-1-epb-10.1177_23998083221133397.pdf]

Table S1. List of the 69 morphometrics, alongside spatial element, scale, spatial context and conceptual category.

character	element	scale	context	category
area	building	S	building	dimension
height	building	S	building	dimension
volume	building	S	building	dimension
perimeter	building	S	building	dimension
courtyard area	building	S	building	dimension
form factor	building	S	building	shape
volume to façade ratio	building	S	building	shape
circular compactness	building	S	building	shape
corners	building	S	building	shape
squareness	building	S	building	shape
equivalent rectangular index	building	S	building	shape
elongation	building	S	building	shape
centroid - corner distance deviation	building	S	building	shape
centroid - corner mean distance	building	S	building	shape
cardinal orientation	building	S	building	distribution
street alignment	building	S	building	distribution
cell alignment	building	S	building	distribution
longest axis length	tessellation cell	S	tessellation cell	dimension
area	tessellation cell	S	tessellation cell	dimension
circular compactness	tessellation cell	S	tessellation cell	shape
equivalent rectangular index	tessellation cell	S	tessellation cell	shape
cardinal orientation	tessellation cell	S	tessellation cell	distribution
coverage area ratio	tessellation cell	S	tessellation cell	intensity
floor area ratio	tessellation cell	S	tessellation cell	intensity
length	street segment	S	street segment	dimension
width	street profile	S	street segment	dimension
height	street profile	S	street segment	dimension

height to width ratio	street profile	S	street segment	shape
openness	street profile	S	street segment	distribution
width deviation	street profile	S	street segment	diversity
height deviation	street profile	S	street segment	diversity
linearity	street segment	S	street segment	shape
area covered	street segment	S	street segment	dimension
buildings per meter	street segment	S	street segment	intensity
area covered	street node	S	street node	dimension
shared walls ratio	adjacent buildings	M	adjacent buildings	distribution
alignment	neighbouring buildings	M	neighbouring cells (queen)	distribution
mean distance	neighbouring buildings	M	neighbouring cells (queen)	distribution
weighted neighbours	tessellation cell	M	neighbouring cells (queen)	distribution
area covered	neighbouring cells	M	neighbouring cells (queen)	dimension
reached cells	neighbouring segments	M	neighbouring segments	intensity
reached area	neighbouring segments	M	neighbouring segments	dimension
degree	street node	M	neighbouring nodes	distribution
mean distance to neighbouring nodes	street node	M	neighbouring nodes	dimension
perimeter wall length	adjacent buildings	L	joined buildings	dimension
mean inter-building distance	neighbouring buildings	L	cell queen neighbours 3	distribution
weighted reached blocks	neighbouring tessellatio cells	L	cell queen neighbours 3	intensity
area	block	L	block	dimension
perimeter	block	L	block	dimension
circular compactness	block	L	block	shape
equivalent rectangular index	block	L	block	shape
compactness-weighted axis	block	L	block	shape
cardinal orientation	block	L	block	distribution
weighted neighbours	block	L	block	distribution
weighted cells	block	L	block	intensity
local meshedness	street network	L	nodes 5 steps	connectivity
mean segment length	street network	L	segment 3 steps	dimension

cul-de-sac length	street network	L	nodes 3 steps	dimension
area covered	street network	L	nodes 3 steps	dimension
reached cells	street network	L	segment 3 steps	dimension
reached cells	street network	L	nodes 3 steps	dimension
reached area	street network	L	nodes 3 steps	dimension
node density	street network	L	nodes 5 steps	intensity
weighted node density	street network	L	nodes 5 steps	intensity
proportion of cul-de-sacs	street network	L	nodes 5 steps	connectivity
proportion of 3-way intersections	street network	L	nodes 5 steps	connectivity
proportion of 4-way intersections	street network	L	nodes 5 steps	connectivity
local closeness centrality	street network	L	nodes 5 steps	connectivity
square clustering	street network	L	nodes within network	connectivity

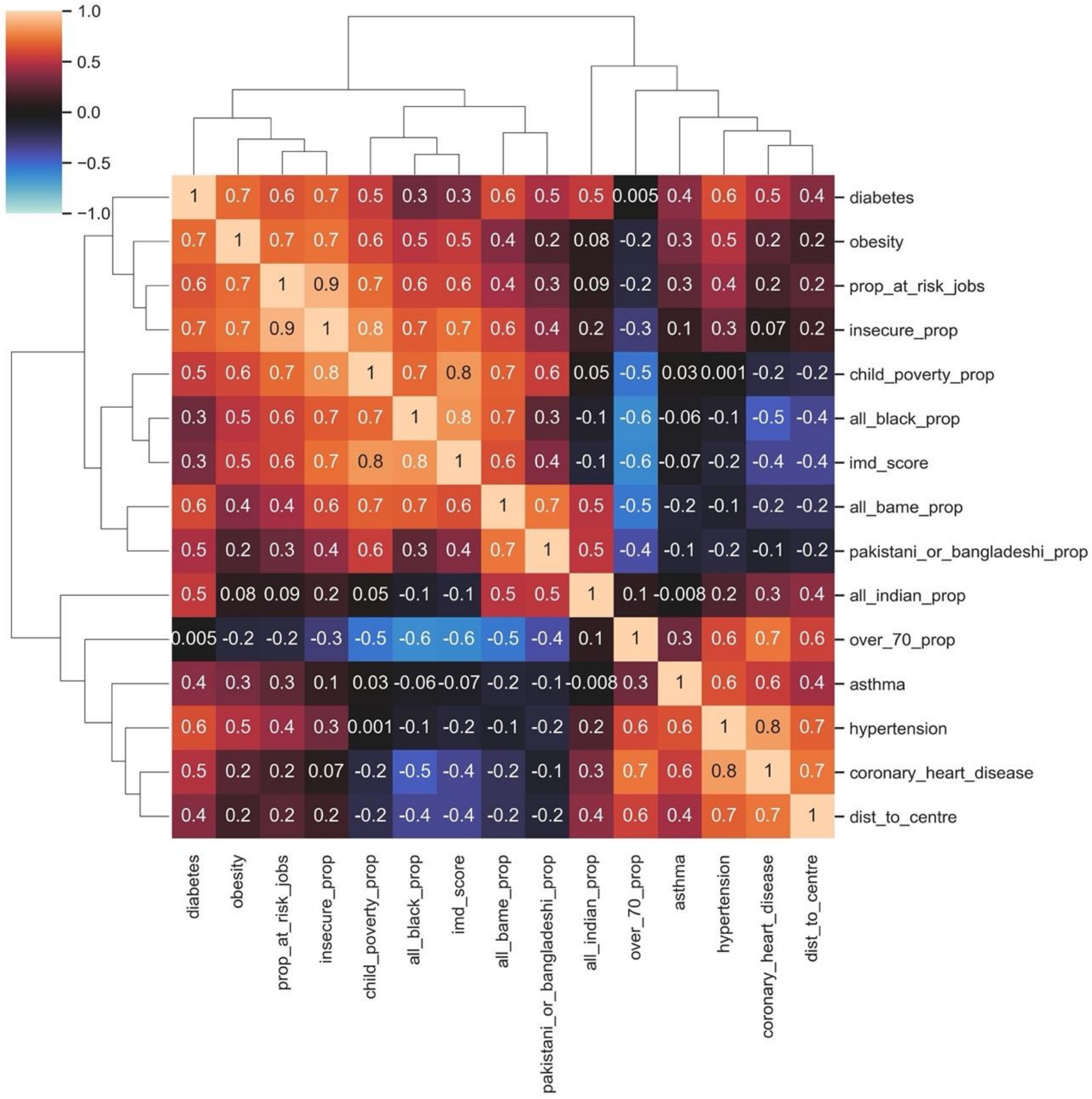

Figure S1. Hierarchically clustered cross-correlation matrix of the control variables. Lighter warm hues correspond to stronger positive correlations. Black

corresponds to no correlation. Lighter cold hues correspond to stronger negative correlations.

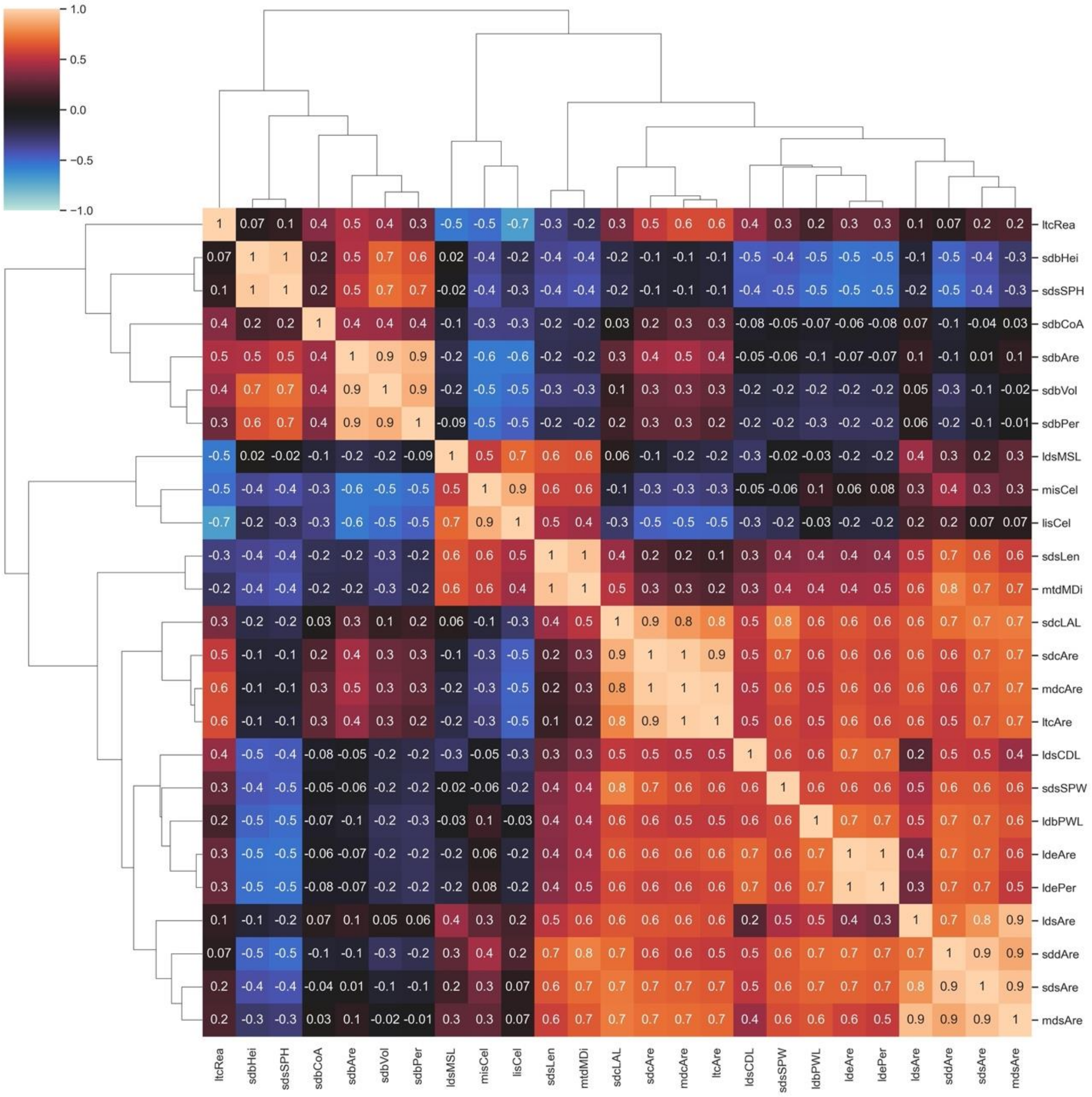

Figure S2. Hierarchically clustered cross-correlation matrix of the morphometrics related to dimension. Lighter warm hues correspond to stronger

positive correlations. Black corresponds to no correlation. Lighter cold hues correspond to stronger negative correlations.

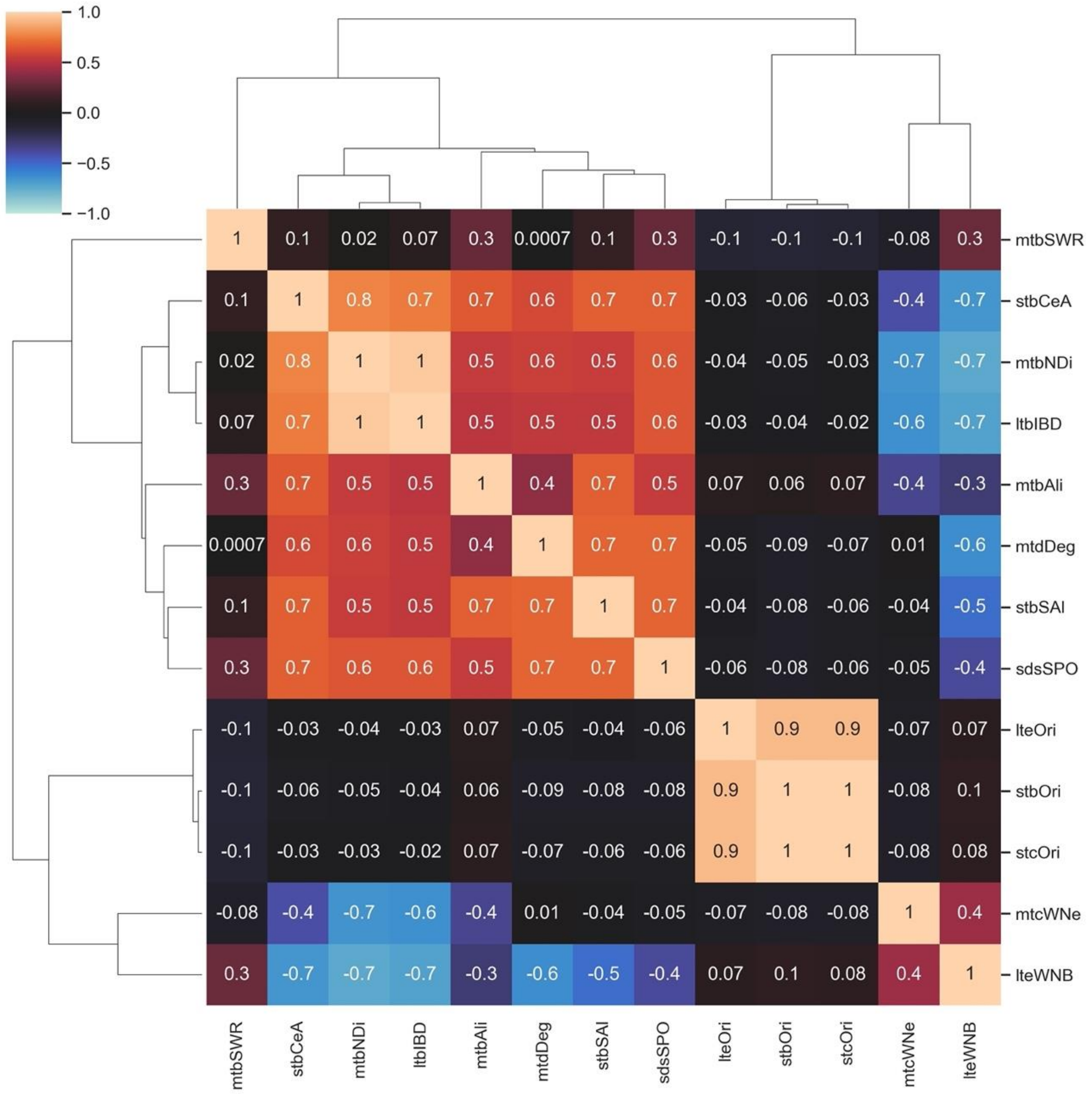

Figure S3. Hierarchically clustered cross-correlation matrix of the morphometrics related to distribution. Lighter warm hues correspond to stronger

positive correlations. Black corresponds to no correlation. Lighter cold hues correspond to stronger negative correlations.

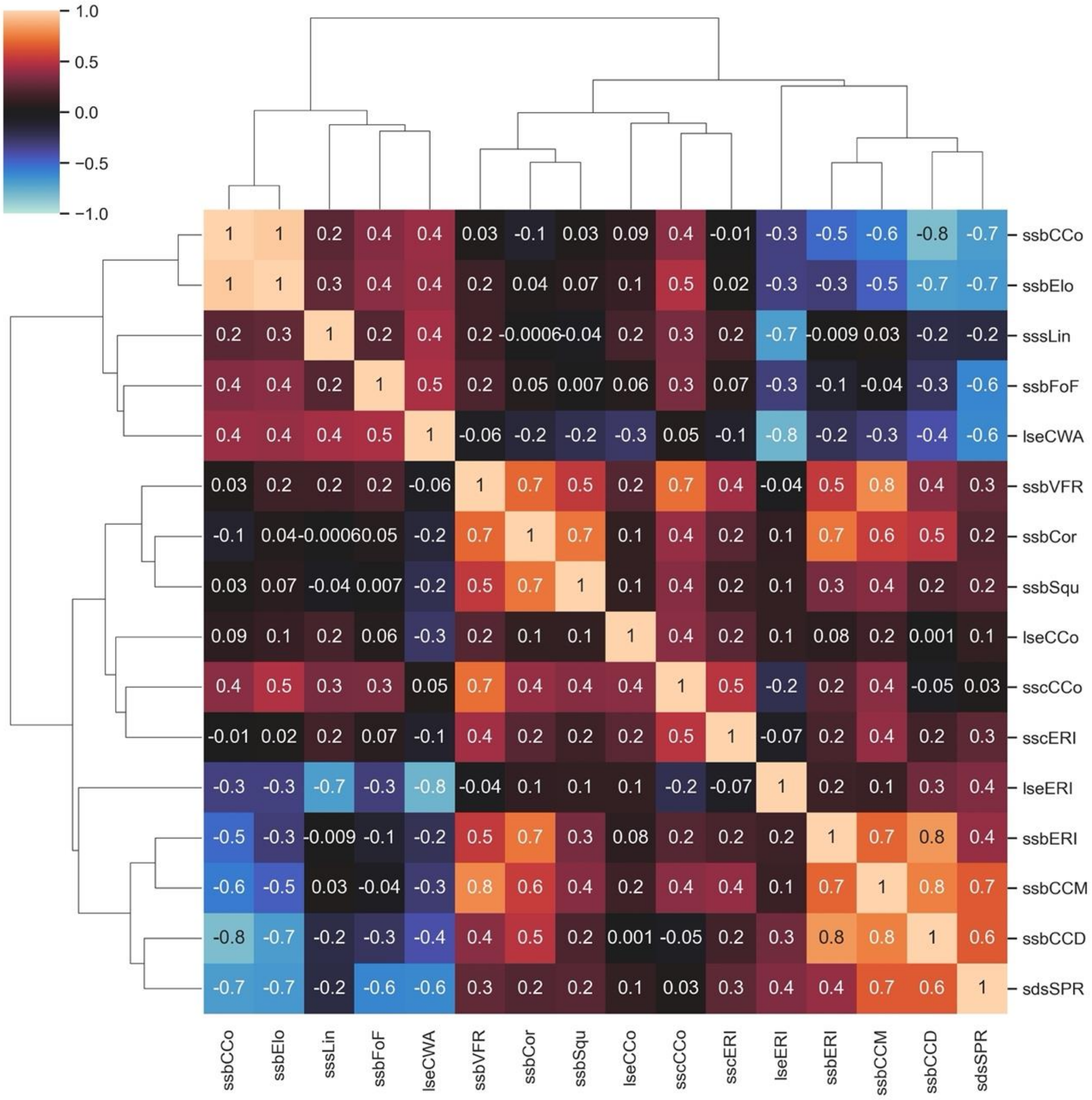

Figure S4. Hierarchically clustered cross-correlation matrix of the morphometrics related to shape. Lighter warm hues correspond to stronger

positive correlations. Black corresponds to no correlation. Lighter cold hues correspond to stronger negative correlations.

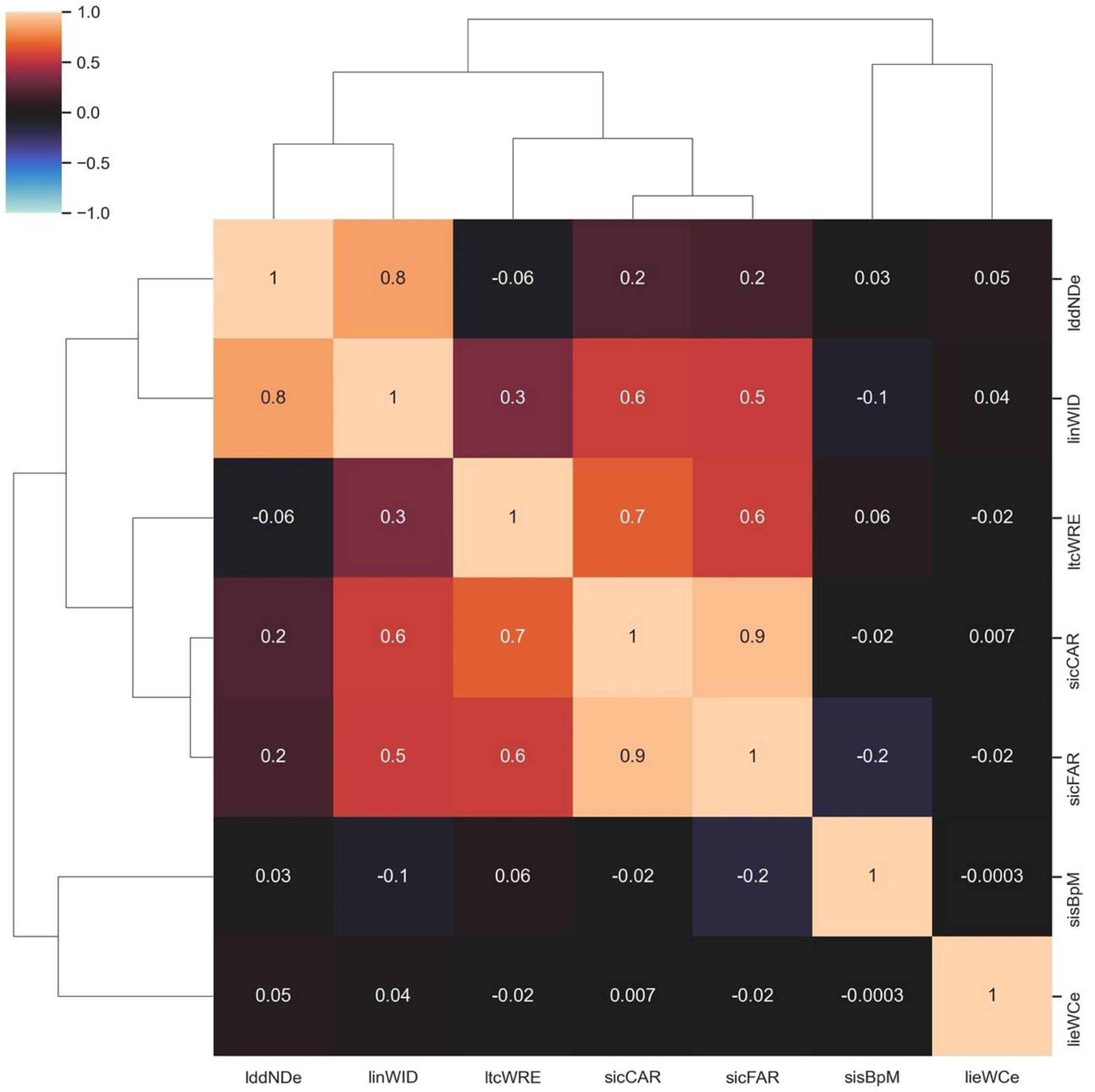

Figure S5. Hierarchically clustered cross-correlation matrix of the morphometrics related to intensity. Lighter warm hues correspond to stronger

positive correlations. Black corresponds to no correlation. Lighter cold hues correspond to stronger negative correlations.

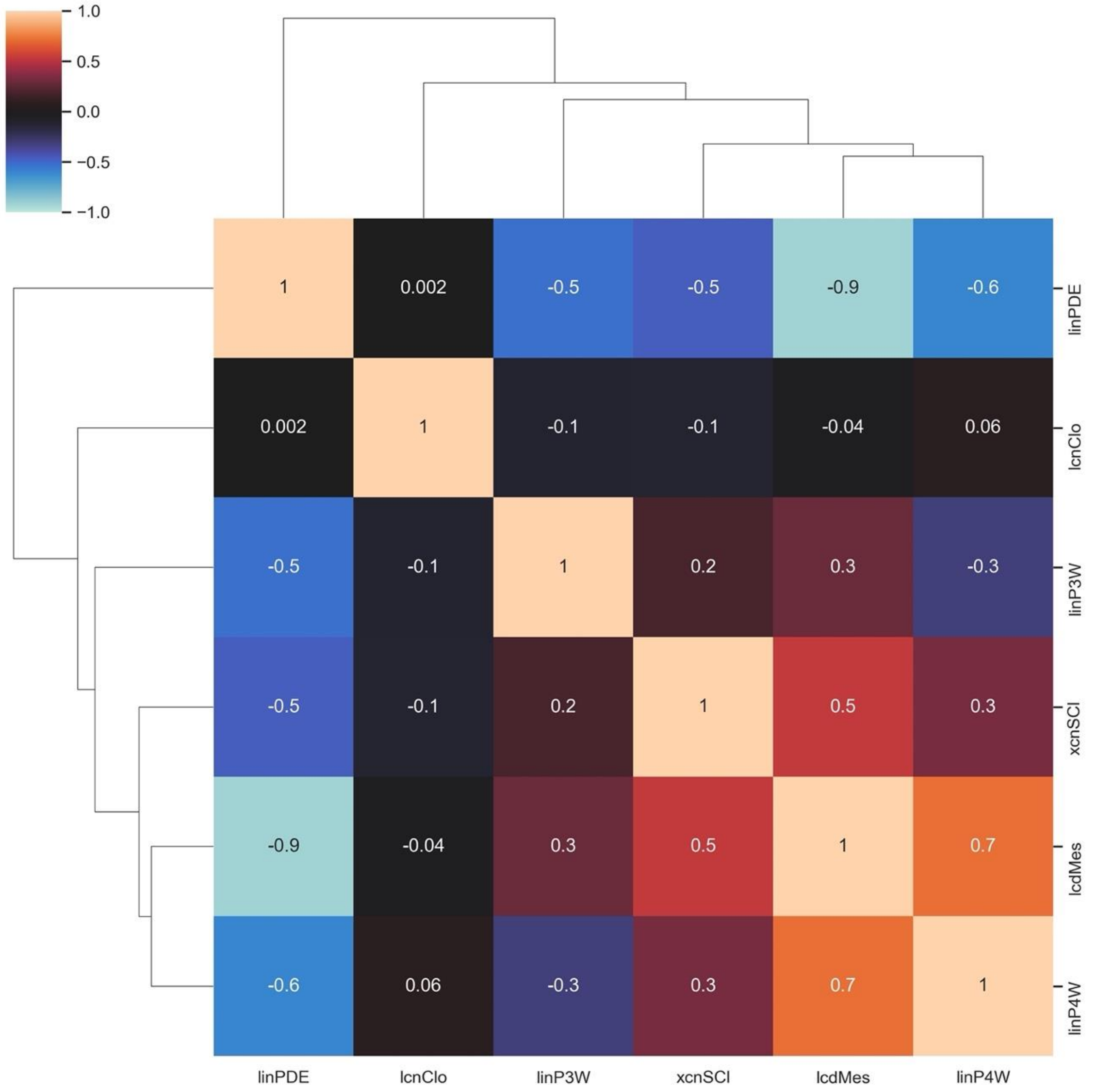

Figure S6. Hierarchically clustered cross-correlation matrix of the morphometrics related to connectivity. Lighter warm hues correspond to

stronger positive correlations. Black corresponds to no correlation. Lighter cold hues correspond to stronger negative correlations.

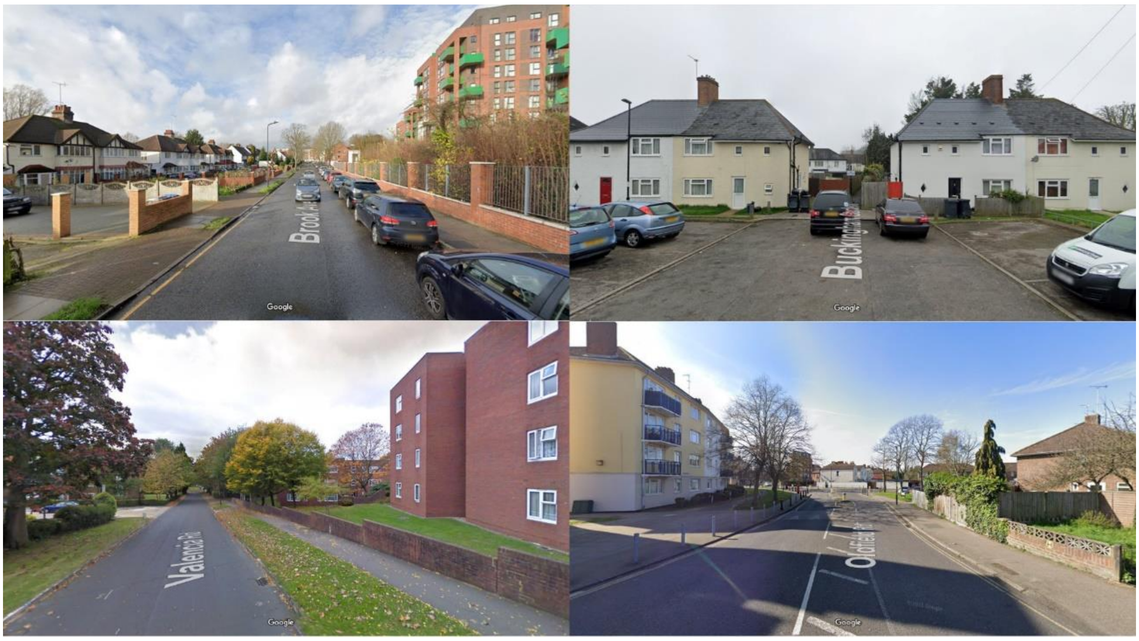

Figure S7. Street views of worst affected MSOAs in terms of COVID-19-related deaths (top row) and cases (bottom row), where models perform the best. Source: Google Street View.

Table S2. Regression model for COVID-19 deaths per 1k residents

Variable	Coefficient	Std.Error	t-Statistic	Probability
CONSTANT	0.000	0.028	0.000	1.000
Proportion of population (PoP) over age 70	0.533	0.043	12.46	0.000
IMD score	0.449	0.038	11.95	0.000
Proportion of population (PoP) with Indian ethnicity	0.216	0.033	6.527	0.000
Distance to centre	-0.076	0.069	-1.096	0.273
Proportion of cul-de-sacs (linPDE)	0.187	0.052	3.567	0.000
Local closeness (lcnClo)	-0.120	0.037	-3.255	0.001
Street profile openness (sdsSPO)	-0.191	0.040	-4.832	0.000
Blocks' granularity (lteWNB)	0.093	0.058	1.594	0.111
Floor area ratio (sicFAR)	-0.197	0.092	-2.144	0.032
Height to width ratio (sdsSPR)	0.141	0.092	1.525	0.128
			R-squared:	0.229
			Adjusted R-squared:	0.222
			Prob(F-statistic):	6.724e-49
			Moran's I:	0.009
			p-value:	0.528

Table S3. Regression model for COVID-19 cases per 100k residents

Variable	Coefficient	Std.Error	z-Statistic	Probability
CONSTANT	-0.001	0.037	-0.015	0.987
Proportion of population (PoP) over age 70	0.410	0.049	8.386	0.000
Proportion of population (PoP) with Black ethnicity	0.432	0.045	9.652	0.000
Proportion of population (PoP) with diabetes	0.238	0.043	5.596	0.000
Distance to centre	0.198	0.069	2.856	0.004
Building volume (sdbVol)				
Blocks' cardinal orientation (lteOri)	0.161	0.042	3.833	0.000
Floor area ratio (sicFAR)	-0.280	0.101	-2.765	0.006
Building elongation (ssbElo)	-0.067	0.051	-1.311	0.190
Height to width ratio (sdsSPR)	0.276	0.083	3.347	0.001
W_residuals	0.298	0.031	9.680	0.000
			Pseudo R-squared:	0.263
